# Neonatal near miss determinants at a maternity hospital for high-risk pregnancy in Northeastern Brazil: a prospective study

**DOI:** 10.1186/s12884-018-2020-x

**Published:** 2018-10-12

**Authors:** Telmo Henrique Barbosa de Lima, Leila Katz, Samir Buainain Kassar, Melania Maria Amorim

**Affiliations:** 1 Health Sciences University of Alagoas (UNCISAL), Maceió, Brazil; 2 Health Sciences, Federal University of São Paulo (UNIFESP), São Paulo, Brazil; 3 Postgraduate Program, Fernando Figueira Institute of Integral Medicine (IMIP), Obstetric Intensive Care Unit, IMIP, Recife, Brazil; 4 Federal University of Campina Grande (UFCG), Campina Grande, Brazil; 5 Maternidade Santa Mônica, Maceió, Brazil

**Keywords:** Neonatal near miss, Neonatal mortality, Fetal death, Maternal near miss

## Abstract

**Background:**

To investigate the associations of maternal variables – sociodemographic, obstetrical and maternal near miss (MNM) variables – with neonatal near miss (NNM) using the new concept of NNM formulated by the Centro Latino-Americano de Perinatologia (CLAP) and the corresponding health indicators for NNM.

**Methods:**

An analytical prospective cohort study was performed at maternity hospital for high-risk pregnancy in Northeastern Brazil. Puerperal women whose newborn infants met the selection criteria were subjected to interviews involving pretested questionnaires.

Statistical analysis was performed with the Epi Info 3.5.1 program using the Chi square test and Fisher’s exact test when appropriate, with a level of significance of 5%. A bivariate analysis was performed to evaluate differences between the groups. All the variables evaluated in the bivariate analysis were subsequently included in the multivariate analysis. For stepwise logistic regression analysis, a hierarchical model was plotted to assess variable responses and adverse outcomes associated with MNM and NNM variables.

**Result*s*:**

There were 1002 live births (LB) from June 2015 through May 2016, corresponding to 723 newborn infants (72.2%) without any neonatal adverse outcomes, 221 (22%) NNM cases, 44 (4.4%) early neonatal deaths and 14 (1.4%) late neonatal deaths. The incidence of NNM was 220/1000 LB. Following multivariate analysis, the factors that remained significantly associated with increased risk of NNM were fewer than 6 prenatal care visits (odds ratio (OR): 3.57; 95% confidence interval (CI): 2.57–4.94) and fetal malformations (OR: 8.78; 95% CI: 3.69–20.90). Maternal age older than 35 years (OR: 0.43; 95% CI: 0.23–0.83) and previous cesarean section (OR: 0.45; 95% CI: 0.29–0.68) protected against NNM.

**Conclusion:**

Based on the large differences between the NNM and neonatal mortality rates found in the present study and the fact that NNM seems to be a preventable precursor of neonatal death, we suggest that all cases of NNM should be audited. Inadequate prenatal care and fetal malformations increased the risk of NNM, while older maternal age and a history of a previous cesarean section were protective factors.

## Background

One of the United Nations (UN) Millennium Development Goals (MDGs) was to reduce the child mortality rate by two-thirds. Brazil met this goal before 2015, with a more considerable reduction in postneonatal mortality and a slower reduction in early neonatal mortality; early neonatal mortality is currently the main component of child mortality [1].

As part of the UN Agenda for Sustainable Development, the goal for the period from 2016 to 2030 is to end preventable deaths of newborns and children under 5 years of age. For this purpose, all the countries that signed the UN document committed to reducing neonatal mortality to at least 12 per 1000 live births (LB) [2].

To assess and improve the quality of the care delivered to this population, reliable assessment instruments are necessary. Child mortality rate has long been used as a classic indicator of social development, economics and healthcare quality [3]. However, for each child who dies, many others survive serious complications; as in the case of maternal health, application of the near miss concept to the neonatal setting might be useful to detect risk factors for death, investigate the quality of care delivered to this population, strengthen the healthcare system and reduce the child mortality rate [4].

However, until recently, there has not been a standardized definition or any international criteria for detecting neonatal near miss (NNM) that would allow performing comparisons within the same institution over time or between different institutions in various regions or countries. In response to this scenario, in 2015, the Latin American Centre of Perinatology (Centro Latino-Americano de Perinatologia - CLAP) led discussions and proposals aiming at establishing a standardized definition of NNM [5] based on the results of previous studies on the subject [4–6]. The CLAP suggests defining NNM as any newborn infant who exhibited pragmatic and/or management criteria and survived the first 27 days of life.

Two studies have described the impact of maternal near miss (MNM) on fetal and neonatal mortality and associated factors and concluded that fetal and neonatal death rates are high among MNM patients [7, 8]. It would seem logical for MNM to be strongly associated with NNM because their determinants – socioeconomic variables, demographic variables, reproductive history, health conditions during pregnancy, prenatal care and labor care – might be associated. However, we were not able to identify any study that investigated the association between these two conditions.

Therefore, the aim of the present study was to investigate the associations of maternal variables – sociodemographic, obstetrical and MNM variables – with NNM using the new concept of NNM formulated by the CLAP and to evaluate the corresponding health indicators for NNM.

## Methods

The present prospective, analytical cohort study was conducted at Santa Monica Maternity School Hospital (Maternidade Escola Santa Mônica - MESM), which is located in Maceió, the capital of the state of Alagoas, in northeastern Brazil. MESM had the lowest Human Development Index (HDI) of 0.631 in the country in 2016. MESM is a public maternity hospital for women with high-risk pregnancies. It is the main obstetrical and neonatal referral center for highly complex cases in Alagoas, and approximately 50% of women with high-risk pregnancies in the state receive care at MESM.

Data collection was performed from June 2015 through May 2016 by the principal investigator and research assistants who were students of an undergraduate medical course and had received specific training for the study. The study was approved by the Human Research Ethics Committee of Universidade Estadual de Ciências da Saúde de Alagoas (UNCISAL) (CAAE no. 37977014.0.0000.5011). All the included women or their legally responsible parties were approached on the day after delivery by the researcher or one of the assistants and invited to participate in the study. They were included only after voluntarily agreeing to participate and signing an informed consent form.

The outcome variable was NNM, and the exposure variables were age, race, marital status, education level, origin, family income, prenatal care, number of prenatal visits, prenatal care performed at the hospital, household visits, current pregnancy, previous cesarean section, referral for childbirth, previous pregnancies, maternal admission to an intensive care unit (ICU), fetal presentation, smoking history, fetal malformations, comorbidities and MNM.

NNM was defined as a neonate who had suffered a life-threatening condition but survived the first 27 days of life [5]. Two sets of criteria that are recommended by the CLAP were used to identify newborn infants at a high risk of death at birth: pragmatic criteria (gestational age at birth less than 33 weeks; birth weight <  1750 g; and 5-min Apgar score < 7) and management criteria (parenteral antibiotics for up to 7 days before 28 days of age; use of a continuous positive airway pressure (CPAP) device; any intubation lasting for up to 7 days before 28 days of age; phototherapy within the first 24 h of life; cardiopulmonary resuscitation; use of vasoactive drugs, anticonvulsants, surfactant, or blood-derived products or use of steroids to treat refractory hypoglycemia; and any surgical procedure) [5].

Some indicators of perinatal care quality were calculated [5]: early neonatal mortality rate (ENMR), neonatal mortality rate (NMR), neonatal near miss rate (NNMR), early severe neonatal outcome rate (ESNOR), severe neonatal outcome rate (SNOR), and lethality ratio.

MNM was defined as a woman who survived a severe, life-threatening complication during pregnancy, childbirth or within 42 days of termination of pregnancy and met any of the clinical, laboratory or management criteria formulated by the World Health Organization (WHO) [9].

Puerperal women whose newborn infants met the selection criteria were subjected to interviews involving pretested questionnaires; other relevant information was obtained from medical records. The data for the newborn infants were collected during the immediate postpartum period and from their medical records. A second interview (by phone call or a home visit) was performed 42 days after childbirth to assess maternal and neonatal outcomes.

Data were collected on printed forms that were stored in the hospital until the final maternal and neonatal outcomes were assessed. Data were then entered into a database created specifically for this purpose in the statistical program Epi Info 3.5.1 (Atlanta, GA), in which the statistical analysis was performed. The data were entered twice and compared, and inconsistencies were corrected. In the bivariate analysis, NNM was the outcome variable, and the aforementioned exposure variables were dichotomized as yes/no variables. The risk ratio (RR) was estimated as a measure of risk, with the corresponding 95% confidence interval (95% CI). The Chi square test or Fisher’s exact test was used as necessary.

All the variables considered for the bivariate analysis were included in a multiple logistic regression analysis to identify the variables that were most strongly associated with NNM, and the adjusted risk was calculated. A hierarchical model was plotted for multiple regression analysis; the variables were included in blocks according to risk categories, and biological and socioeconomic variables including race/skin color (nonwhite), educational level (less than 8 years of schooling), origin (inner state), income (less than the minimum wage), age (younger than 20 years old), age (older than 35 years old) and marital status (without a partner) were the most distal factors. In the intermediate level, variables corresponding to prenatal and labor care were included: no prenatal care, fewer than 6 prenatal care visits, prenatal care not performed at the hospital where the study was conducted, no referral to a maternity hospital for childbirth, previous cesarean section, nulliparity, noncephalic presentation, maternal admission to an ICU and smoking history. Variables that were considered to be closest to the outcome of NNM were included in the proximal level: comorbidities, MNM (clinical, laboratory and management criteria) and fetal malformations.

Stepwise logistic regression was performed; at the end of each block, the variables associated with the outcome at a 20% significance level were selected, followed by those that remained associated with the outcome at a 5% significance level. A final regression analysis was then performed to determine the adjusted risk for NNM for each of the variables that were significantly associated with the outcome at a 5% significance level. Odds ratios (ORs) and corresponding 95% CIs were calculated for these variables.

## Results

**Fig. 1:**
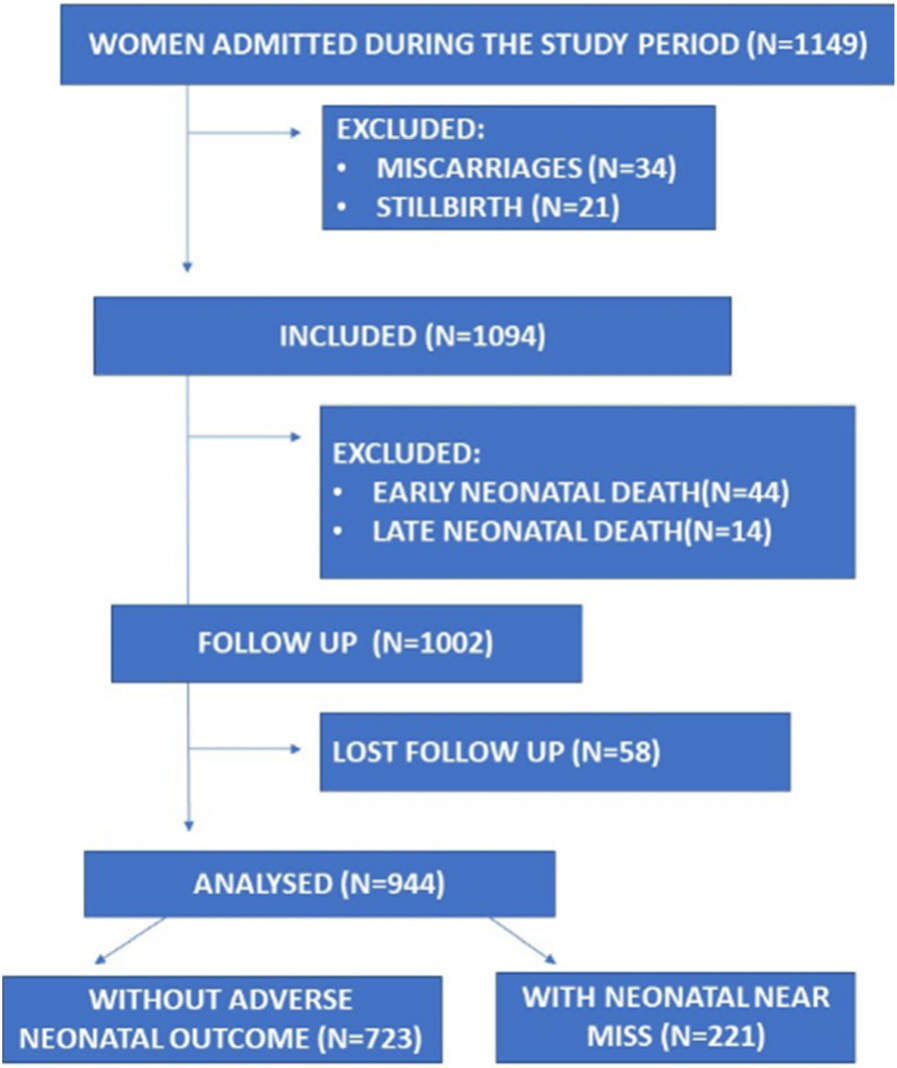
Patient’s Flowchart

From June 2015 to May 2016, 1149 women were admitted to the maternity ward. Thirty-four had miscarriages, and 21 delivered stillborn infants. The initial interview was performed with 1094 women on the first postpartum day, and when they were invited, all of them consented to participate. After initial inclusion, during the follow-up period, 44 newborns (4.4%) were early neonatal deaths, and 14 (1.4%) were late neonatal deaths. Of the 1002 LB that could be analyzed, 58 newborns were lost to follow-up; thus, 723 babies that did not exhibit any neonatal adverse outcomes (72.2%) and 221 (22%) NNM cases were included (Fig. 1).

Indicators of perinatal care quality were calculated for the aforementioned period, resulting in 220 NNM cases/1000 LB, a SNOR of 278/1000 LB and an NMR of 57/1000 LB (Table 1).

**Table 1:** Indicators of perinatal outcomes

Indicators	No.	Rate
Number of live births	1002	
Early neonatal deaths	44	
Late neonatal deaths	14	
Newborn infants with neonatal near miss	221	
Neonatal near miss rate		220/1000 LB
Early severe neonatal outcome rate		264/1000 LB
Severe neonatal outcome rate		278/1000 LB
Early neonatal mortality rate		44/1000 LB
Neonatal mortality rate (early + late)		57/1000 LB
Lethality ratio	3.8	

A total of 131 newborn infants met the pragmatic criteria for NNM, corresponding to an incidence of 131/1000 LB. Among the pragmatic criteria, gestational age younger than 33 weeks (47.5%) was the most commonly identified criterion, with an incidence of 105/1000 LB. All 221 newborn infants met the management criteria, corresponding to an incidence of 220/1000 LB; the use of a nasal CPAP device was the most commonly identified criterion (62.9%), with an incidence of 139/1000 LB (Table 2). Ninety newborn infants presented only the management criteria, while the remaining infants had both pragmatic and management criteria.

**Table 2:** Criteria for neonatal near miss

Criteria	No.	%	Incidence of neonatal near miss/1000 LB
Pragmatic criteria	131	59.3	131.2
Gestational age < 33 weeks	105	47.5	105.2
Weight (< 1750 g)	99	44.8	99.1
5-min Apgar score (< 7)	15	6.8	15.0
Management criteria	221	100.0	220.0
Nasal continuous positive airway pressure	139	62.9	139.2
Surfactant	100	45.2	100.2
Intubation	84	38.0	84.1
Parenteral antibiotics	67	30.3	67.1
Blood-derived products	41	18.6	41.0
Phototherapy during the first 24 h	22	10.0	22.0
Vasoactive drugs	11	5.0	11.0
Surgical procedures	11	4.9	11.0
Anticonvulsants	7	3.2	7.0
Cardiopulmonary resuscitation	05	2.3	5.1
Steroids for hypoglycemia	01	0.5	1.0

In the hierarchical bivariate analysis, the sociodemographic, obstetrical and MNM variables that exhibited statistically significant associations with NNM were age younger than 20 years (RR = 1.58; 95% CI = 1.25–1.99), age older than 35 years (RR = 0.37; 95% CI = 0.21–0.64), fewer than 6 prenatal care visits (RR = 2.63; 95% CI = 2.08–3.33), no referral for childbirth (RR = 1.49; 95% CI = 1.15–1.94), history of a previous cesarean section (RR = 0.52; 95% CI = 0.37–0.73), nulliparity (RR = 1.31; 95% CI = 1.04–1.65), noncephalic presentation (RR = 1.78; 95% CI = 1.30–2.44), maternal admission to an ICU (RR = 1.64; 95% CI = 1.17–2.28), fetal malformations (RR = 3.07; 95% CI = 2.32–4.08), MNM (RR = 1.94; 95% CI = 1.31–2.89), clinical criteria for MNM (RR = 1.99; 95% CI = 1.32–2.98) and management criteria for MNM (RR = 1.86; 95% CI = 1.12–3.1) (Table 3).

**Table 3:** Associations of sociodemographic and obstetrical variables and maternal near miss with neonatal near miss

	Neonatal near miss		RR (95% CI)	*P*
	Yes, n (%)	No, n (%)		
Distal factors				
Race/skin color (nonwhite)	170(76.9)	583(80.6)	0.84 (0.64–1.10)	0.22
Education level (< 8 years)	121(54.8)	385(53.3)	1.04 (0.83–1.32)	0.69
Origin (inner state)	116(52.5)	359(49.7)	1.09 (0.86–1.37)	0.46
Income (< minimum wage)	185(83.7)	571(79.0)	1.27 (0.92–1.75)	0.12
Age (< 20 years old)	86(38.9)	185(25.6)	1.58 (1.25–1.99)	0.0001
Age (> 35 years old)	12(5.4)	114(15.8)	0.37 (0.21–0.64)	0.00007
Marital status (without a partner)	54(24.4)	141(19.5)	1.24 (0.95–1.61)	0.11
Intermediate factors				
No prenatal care	6(2.7)	11(1.5)	1.52 (0.79–2.92)	0.18^*^
< 6 prenatal care visits	133(60.2)	211(29.2)	2.63 (2.08–3.33)	< 0.0000001
Prenatal care at service	179(81.0)	544(75.2)	1.30 (0.96–1.75)	0.07
No household visits	219(99.1)	698(96.5)	3.22 (0.84–12.29)	0.04
No referral for childbirth	160(72.4)	441(61.0)	1.49 (1.15–1.94)	0.002
Current pregnancy (cesarean section)	165(74.7)	548(75.8)	0.95 (0.73–1.24)	0.73
Previous cesarean section	35(15.8)	214(29.6)	0.52 (0.37–0.73)	0.00004
Nulliparity	112(50.7)	302(41.8)	1.31 (1.04–1.65)	0.01
Noncephalic presentation	28(12.7)	43(6.0)	1.78 (1.30–2.44)	0.0009
Admission to an intensive care unit	25(11.3)	43(5.9)	1.64 (1.17–2.28)	0.006
Smoker	9(4.2)	20(2.7)	1.33 (076–2.33)	0.32
Proximal factors				
Fetal malformation	19(8.6)	9(1.2)	3.07 (2.32–4.08)	< 0.0000001
Comorbidities	78(35.3)	306(42.3)	0.79 (0.62–1.01)	0.06
Maternal near miss	15(6.7)	19(2.6)	1.94 (1.31–2.89)	0.003
Maternal near miss clinical criteria	14(6.3)	17(2.3)	1.99 (1.32–2.98)	0.003
Maternal near miss laboratory criteria	05(2.2)	9(1.2)	1.53 (0.75–3.13)	0.42^*^
Maternal near miss management criteria	09(4.0)	12(1.7)	1.86 (1.12–3.1)	0.07^*^

^*^Analysis performed with Fisher’s exact test

All the variables assessed in the bivariate analysis were included in a multivariate analysis involving a logistic regression model with hierarchical levels. The factors that remained significantly associated with a higher risk of NNM were fewer than 6 prenatal care visits (OR = 3.57; 95% CI = 2.57–4.94) and the presence of fetal malformations (OR = 8.78; 95% CI = 3.69–20.90), while maternal age older 35 years (OR = 0.43; 95% CI = 0.23–0.83) and history of a previous cesarean section (OR = 0.45; 95% CI = 0.29–0.68) were protective against NNM (Table 4).

**Table 4:** Multivariate analysis of neonatal near miss determinants

	OR	95% CI	*P*
Distal factors			
Age (> 35 years old)	0.43	0.23–0.83	0.0118
Intermediate factors			
Previous cesarean section (yes)	0.45	0.29–0.68	0.0002
< 6 prenatal care visits	3.57	2.57–4.94	0.0000
Proximal factors			
Fetal malformation (yes)	8.78	3.69–20.90	0.0000

## Discussion

In this study, the incidence of NNM was 220/1000 LB. Following multivariate analysis, the factors that remained significantly associated with a higher risk of NNM were fewer than six prenatal care visits and the presence of fetal malformations, while maternal age older than 35 years and previous cesarean section were protective factors against NNM.

We conducted an active search of the PubMed, Biblioteca Regional de Medicina (BIREME) and Scopus databases. This study is the first to analyze the association between MNM and the severe neonatal condition NNM with prospectively collected data and the use of the 2015 CLAP criteria [5] to define NNM in an attempt to standardize the definition of NNM and to facilitate comparisons of NNM rates between different regions and countries.

In our study, NNM accounted for 79.2% of the adverse perinatal outcomes; this NNM rate was 3.8 times higher than the NMR. In other studies, the NNM rate varied from 2.6- to 8-fold higher than the NMR, even when the same criteria and markers were used [4, 6, 10, 11].

The high NNM rate (220/1000 LB) and NMR (57/1000 LB) found in the present study differ from those in other reports (21.4–72.5/1000 LB and 6.3–11.1/1000 LB, respectively) [4, 6, 10, 11]. These differences might be attributable to the fact that the present study considered the associations of pragmatic (59.3%) and management (40.7%) criteria. Several studies have shown that the combination of these criteria exhibits better performance than either set of criteria used alone, with sensitivities and specificities of almost 93% [5, 12]; the use of this combination of criteria allowed us to assess a larger number of surviving newborn infants. Another reason that might explain the high NNM rate and NMR found in the present study is that other authors employed population databases [4, 6, 10, 11], while the present study used only data from a maternity hospital that is a referral center for women with high-risk pregnancies; this sample selection was a source of bias that resulted in high frequencies of adverse outcomes. In addition, the observation period in our study was 28 days, while in other studies, it varied from 3 to 28 days after birth [4, 6, 10, 11] thus limiting the assessment of the quality of late neonatal care and neonatal mortality. By selecting a prospective design in which neonatal complications were monitored on a daily basis, we possibly avoided the losses that might occur in studies involving retrospective databases. Finally, the high NNM rate and NMR found might be related to the socioeconomic status of the state of Alagoas, which has the lowest HDI in Brazil.

Currently, comparison of the NNM rates of different hospitals is difficult due to the lack of a universally accepted definition of NNM, as there is for MNM [9]. In response to this scenario, in 2015, the CLAP guided a series of discussions and proposals to develop a consensus definition of NNM based on pragmatic and management criteria [5].

The management criteria for NNM cannot be used to compare the quality of healthcare between different hospitals because the NNM rate might be influenced by the technological complexity of the services provided [12], unless a general complexity index is developed and included in the statistical analysis [11]. Thus, the best option to detect NNM cases in hospitals with more substantial resources, such as ICUs and neonatal ICUs, as in the maternity hospital where the present study was conducted, is to combine the pragmatic and management criteria [5, 12].

As NNM is known to be a useful assessment of the quality of obstetrical and neonatal care [4–6, 10, 11], we sought to investigate whether maternal variables (sociodemographic, obstetrical and MNM variables) were related to NNM.

Multivariate analysis provided two results that diverged from those of other reports in the literature in terms of age extremes. First, age younger 20 years did not exhibit an association with NNM, although adolescent pregnancy is usually associated with poorer maternal and perinatal outcomes including prematurity, low birth weight and occurrence of 5-min Apgar scores of less than 7, than pregnancy in adult women [3, 13–15]. Other mechanisms that are not biological might mediate the relationships between adolescence and adverse obstetric/perinatal outcomes, which previous studies failed to detect.

Age older than 35 years exhibited a statistically significant association with NNM in the multivariate analysis, demonstrating evidence of a protective effect. This finding is in clear contrast with those of previous reports on age extremes and perinatal outcomes [3, 15]. The data on the risk associated with pregnancy at an age older than 35 years are conflicting due to the presence of other variables, such as parity and pre-existing diseases, which impair assessment of the risk associated with maternal age alone [16]. However, in the present study, we ruled out confounding variables; thus, the results might be explained by the fact that currently, most pregnancies among women older than 35 years are planned, and prenatal and required care are prioritized, leading to consequent improvement in perinatal outcomes. Alternatively, when caring for older pregnant women with or without associated diseases, healthcare providers might be more alert and intervene earlier in these women than in younger pregnant women, thus preventing adverse outcomes. Therefore, the results described here might pave the way for new studies.

Prenatal care is important. In theory, more prenatal visits might mean higher odds of receiving care, especially in the case of high-risk pregnancy, which is associated with poor maternal and perinatal outcomes. In Brazil, the prenatal care coverage and number of visits have increased over the past 15 years [17]. However, studies conducted in the country have still revealed flaws in prenatal care, such as difficult access to care, late onset of care, an inadequate number of visits and incomplete performance of procedures, all of which impact perinatal outcomes [18–20]. Such outcomes are below the country’s potential and reflect unfavorable living and healthcare conditions, in addition to historical regional and socioeconomic inequalities [3].

Our findings confirm the deleterious effects of inadequate prenatal care. Attending fewer than 6 prenatal care visits was statistically significantly associated with NNM, increasing the risk of NNM by 4-fold. The nationwide hospital-based survey Birth in Brazil, which was conducted in 2014, analyzed neonatal mortality profiles and found high NNM rates among mothers who received inadequate prenatal care [3]. The underlying causes of most neonatal deaths are maternal obstetrical problems that were not resolved prenatally [21]. Therefore, the prenatal care coverage needs to be broadened, and the quality of the care delivered improved, especially for women with high-risk pregnancies.

Women with a history of a previous cesarean section are more often subjected to a second cesarean section because many obstetricians still base their decision-making on a principle that was formulated in 1916, according to which “once a C-section, always a C-section,” and fear rupture of uterine scars [22]. However, there is no scientific evidence supporting this alleged risk, as uterine scar rupture occurs in approximately 1% of patients [23]. In our study, 94.3% of women with a history of a previous cesarean section were again subjected to cesarean section. In the multivariate analysis, this variable was protective against NNM. The presence of a previous uterine scar might have led to the decision to perform a cesarean section early in the course of labor, and some of the newborn infants might have benefited from the fact that 33.9% of the mothers had high-risk pregnancies that required urgent resolution. Adverse events derived from the lack of rigorous monitoring during labor, which is essential in women with a history of a previous cesarean section, might have been avoided; thus, cesarean section was a protective factor because maternal complications that contribute to the occurrence of adverse perinatal outcomes were prevented.

In one study published in 2017, the survival of newborn infants born to mothers without severe complications was better than that of infants born to mothers with eclampsia, intrapartum hemorrhage or other complications requiring ICU admission [15]. In the multivariate analysis performed in the present study, maternal ICU admission did not remain associated with NNM. Perhaps this marker of maternal severity is not always a marker of neonatal severity and vice versa. For instance, conditions such as puerperal sepsis or hemorrhage do not affect the newborn infant, even though the mother needs to be admitted to an ICU; in the case of spontaneous premature labor, the infant might be an NNM case, although the mother is not an MNM case.

Newborn infants with congenital malformations exhibited a 9-fold increased risk of NNM; this result remained unchanged after controlling for confounding variables. This finding was expected because fetal malformations increase neonatal morbidity and mortality. The literature shows that despite high-quality interventions, in most of these infants, neonatal death due to several fetal malformations cannot be avoided [12]. This variable has been rarely investigated in previous studies [5, 12] and seems to be a relevant marker for characterizing NNM cases; thus, it should be employed in future studies as a defining criterion for NNM.

Studies on MNM are abundant in the literature [24, 25], especially after 2009, following a publication on the definition of and criteria for MNM formulated by the WHO [9]. However, we were not able to locate any study reporting an association between MNM and NNM.

Two studies investigated the relationship between MNM and adverse perinatal outcomes (fetal and neonatal deaths) [7, 8]. In the first study performed in 2013, the authors found high frequencies of fetal and neonatal deaths among MNM cases, and the main factors associated with outcomes were severe pre-eclampsia, placental abruption, prematurity and endometritis; there was also an association between laboratory criteria and adverse outcomes [7]. These results were corroborated by a second study that was performed in 2017 [8].

It would seem logical for NNM to be strongly associated with MNM because the determinants (socioeconomic variables, demographic variables, reproductive history, health status during pregnancy, prenatal care and labor care) might also be associated. However, the multivariate analysis did not reveal an association between MNM variables and NNM outcomes. A possible explanation is that many of the conditions that lead to the classification of a woman as an MNM case are so severe that they lead to early resolution of pregnancy thus sparing the fetus from complications. Alternatively, many of the conditions for which a woman is classified as an MNM case, such as puerperal hemorrhage and sepsis, occur after birth, again sparing the fetus from complications.

Among the strengths of the present study are its prospective cohort design and the fact that a multiple regression model that accounted for the mutual interrelationships of variables was used; thus, potential confounding variables were controlled. In addition, this is one of the first studies to employ the CLAP criteria to define NNM and to investigate the impact of maternal factors on NNM and the association between MNM and NNM, both of which have rarely been analyzed in the literature.

Some limitations of the present study are derived from the fact that NNM was assessed at a hospital for women with high-risk pregnancies; thus, the study sample is not representative of the population of pregnant women in Alagoas. In addition, the data corresponding to socioeconomic variables and access to healthcare were collected in interviews conducted with the participants and might be subject to recall bias.

## Conclusion

Based on the large differences between the NNM rate and NMR found in the present study and the fact that NNM seems to be a preventable precursor of neonatal death, we suggest that all cases of NNM should be audited. Inadequate prenatal care and fetal malformations increased the risk of NNM, while older maternal age and a history of previous cesarean section were protective factors.

## Abbreviations

CLAP: Latin American Centre of Perinatology (Centro Latino-Americano de Perinatologia)
ENMR: Early neonatal mortality rate
ESNOR: Early severe neonatal outcome rate
HDI: Human Development Index
ICU: Intensive care unit
LB: Live births
MDGs: Millennium Development Goals
MESM: Santa Monica Maternity School Hospital (Maternidade Escola Santa Mônica)
MNM: Maternal near miss
NMR: Neonatal mortality rate
NNM: Neonatal near miss
NNMR: Neonatal near miss rate
OR: Odds ratio
RR: Risk ratio
SNOR: Severe neonatal outcome rate
SUS: Unified Heath System (Sistema Único de Saúde)
UN: United Nations
UNCISAL: State University of Health Sciences of Alagoas (Universidade Estadual de Ciências da Saúde de Alagoas)
WHO: World Health Organization
